# The Protective Role of Celastrol in Renal Ischemia-Reperfusion Injury by Activating Nrf2/HO-1, PI3K/AKT Signaling Pathways, Modulating NF-κb Signaling Pathways, and Inhibiting ERK Phosphorylation

**DOI:** 10.1007/s12013-022-01064-6

**Published:** 2022-02-14

**Authors:** Nancy S. Younis, Amal M. H. Ghanim

**Affiliations:** 1 Department of Pharmaceutical Sciences, College of Clinical Pharmacy, King Faisal University, Al-Ahsa, Kingdom of Saudi Arabia; 2 Department of Biochemistry, Faculty of Pharmacy, Fayoum University, Fayoum, Egypt

**Keywords:** Celastrol, Renal Ischemia-Reperfusion, Nrf2/HO-1, PI3K/AKT, ERK phosphorylation

## Abstract

Celastrol, a natural triterpenoid derived from Tripterygium wilfordii, possesses numerous biological effects. We investigated celastrol’s antioxidant potential through nuclear factor erythroid 2-related factor 2 (Nrf2)/heme oxygenase 1 (HO-1) and its effect on phosphoinositide 3-kinase (PI3K)/protein kinase B (AKT) signaling, nuclear factor-kappa B (NF-κB) pathways, and extracellular signal-regulated kinase (ERK) activation in kidney ischemia-reperfusion injury (IRI) rat model. Rats were given celastrol 2 mg/kg orally for 1 week before subjection to renal ischemia-reperfusion surgery. Kidney functions, renal MDA, and reduced glutathione were determined; also, renal levels of ERK1/2, HO-1, PI3K, IL-6, TNF-α, IκBα, NF-κB/p65, and cleaved caspase-3 were measured. In addition, gene expression of kidney injury molecule-1 (KIM-1), Nrf-2, and AKT were determined. Celastrol pretreatment attenuated oxidative stress and increased Nrf2 gene expression and HO-1 level. Also, it activated the PI3K/AKT signaling pathway and decreased the p-ERK:t- ERK ratio and NFκBp65 level, with a remarkable decrease in inflammatory cytokines and cleaved caspase-3 levels compared with those in renal IRI rats. Conclusively, celastrol showed a reno-protective potential against renal IRI by suppressing oxidative stress through enhancing the Nrf2/HO-1 pathway, augmenting cell survival PI3K/AKT signaling pathways, and reducing inflammation by inhibiting NF-κB activation.

## Introduction

**Fig. 1:**
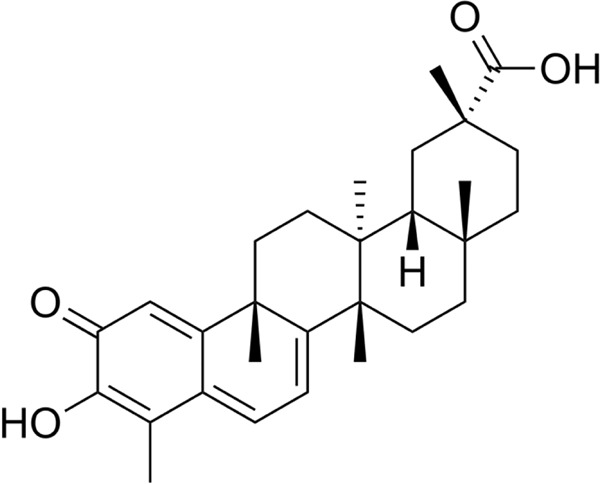
Chemical structure of celastrol

Pharmacological bioactive molecules obtained from medicinal plants have attracted substantial importance lately due to their robust, unique, diverse actions. Celastrol is a pentacyclic triterpene extracted from Tripterygium wilfordi (TW) [1], with the chemical structure provided in Fig. 1. Celastrol attains numerous pharmacological actions, including antioxidant and anti-inflammatory [2, 3]. It has been proven that celastrol suppresses several proinflammatory cytokines, including tumor necrosis alpha (TNF-α), interleukins (IL-2, IL-8), interferon-gamma (IFN-γ), proinflammatory enzymes, nitric oxide synthase, nuclear factor-kappa B (NFκB), and adhesion molecules [4, 5]. Thus, it showed promising actions in diverse inflammatory diseases such as rheumatoid arthritis [6], Crohn’s disease [7], ulcerative colitis [8], and asthma [9]. Additionally, celastrol was proven to possess anti-obesity [10], neuroprotective effect [11] and anti-diabetic [12]. Moreover, Celastrol’s anticancer potential was confirmed in many studies by promoting apoptosis and inhibiting angiogenesis, proliferation, and cell invasion [13–15]. Due to such diverse pharmacological actions, numerous clinical trials were conducted using TW plant extract. Furthermore, randomized clinical trials on patients with various diseases, such as rheumatoid arthritis [16], solid tumor [17], diabetic nephropathy [18], and renal transplantation [19] in attempts to evaluate celastrol therapeutically for a more in-depth investigation on Castrol’s actions on the kidney. A study by Zhang, Chen [20] showed that celastrol reduces blood contents of creatinine and urea nitrogen in a diabetic nephropathy rat model and reduces urinary protein excretion, improving pathological renal injury by regulating mitogen-activated protein kinase (MAPK)/NF-κB pathway. Another research group proved that celastrol treatment could attenuate unilateral ureteral obstruction induced mouse renal fibrosis and folic acid-induced mouse renal fibrosis [21].

In cisplatin-induced kidney injury, Celastrol treatment improved renal function, kidney morphology, oxidative stress, and suppressed renal tubular injury markers; kidney injury molecule (KIM-1) and neutrophil gelatinase-associated lipocalin NGAL). Moreover, it inhibited renal apoptosis and inflammation by deterring tubular cell apoptosis and NF-κB activation and enhancing mitochondrial function [22]. Furthermore, Celastrol improved ischemia-reperfusion induced injury (IRI) as it significantly suppressed renal function markers, oxidative stress and prevented the expression of proinflammatory mediators, which was associated with the suppression of nuclear translocation of NF-κB subunit p65 thus, alleviating renal tubular damage [23]. In this study, we aimed at investigating the antioxidant potential of celastrol through its effect on the nuclear factor erythroid 2-related factor 2 (Nrf2)/heme oxygenase 1 (HO-1) pathway. This pathway is considered a key regulator of cellular antioxidant defense mechanism that may be a good target for new therapeutic approaches to alleviate kidney cell damage induced by IR and the accompanying oxidative stress. Likewise, our study attempted underlining celastrol effect on cell survival mediating pathway; phosphoinositide 3-kinase (PI3K)/protein kinase B (AKT). In addition, exploring Celastrol’s anti-inflammatory and antiapoptotic effects through its effect on extracellular signal-regulated kinase (ERK) as a MAPK family member and NF-κB transcription factor is greatly associated with inflammation and apoptosis during renal injury.

## Material and Methods

### Animals

Forty-eight-week-old adult male Swiss albino rats weighing 200 ± 20 g were obtained from the Faculty of Pharmacy, Delta University, Egypt. The rats were accommodated in a controlled environment (24 ± 1 °C, 50 ± 10 humidity) and a 12 h:12 h light:dark cycle with free access to food and water ad lib. Rat care and experimental techniques were permitted and approved by the Institutional Animal Care and Use Committee (IACUC) at the Faculty of Pharmacy, Delta University for Science and Technology. Ethical approval number: FPDU 6/2019. All experiments were conducted following relevant guidelines and regulations.

### Experimental Design and Surgery

Rats were randomized into four groups (*n* = 10 each). First, sham-operated group, in which rats were subjected to bilateral renal artery separation with no clamping (Sham). Second, sham-celastrol-operated group, in which rats were given celastrol 2 mg/kg, orally [22] for 1 week, then subjected to bilateral renal artery separation with no clamping (Sham + cela). Third, renal IR group, in which kidney injury was induced in rats by renal ischemia-reperfusion surgery (IRI). Finally, renal IR + celastrol group, in which animals were administered celastrol 2 mg/kg orally for 1 week before renal IR surgery (IRI + cela). Animals were anesthetized with 2% pentobarbital sodium (50 mg/kg, i.p.) and maintained on a heating pad to sustain rats’ body temperature at 37 °C to perform renal IR surgery. Then, nephrectomy of the left kidney was performed, and clamping the right bilateral renal artery using a non-traumatic artery clamp for 30 min followed by 24 h reperfusion to induce renal IRI [24].

Twenty-four hours after renal reperfusion, animals were sacrificed by decapitation under anesthesia, and blood samples were collected from different experimental groups. Serum samples were obtained via 15 min of blood centrifugation at 5000 rpm and were stored at −20 °C for subsequent measurements. The right kidney was rapidly removed and separated into two parts; the first part was homogenized and kept at −80 °C for biochemical analyses. The second part was kept in 10% formalin for histopathological examination.

### Assessing Kidney Function and Oxidative Stress

Kidney function was assessed by measuring serum creatinine (Cat. No; 235 001) and blood urea nitrogen (BUN) (Cat. No; 310 100) levels using a commercially available kit obtained from Spectrum Diagnostics, Egypt. For oxidative stress assessment, renal lipid peroxidation assay was performed by measurement of MDA level in kidney tissue homogenate by commercially available kit (Cat. no; LIP39-K01) purchased from Eagle Biosciences, Inc. (Boston, MA, USA). Also, renal reduced glutathione (GSH) assay was performed using the kit attained from BioVision incorporated (Milpitas, CA, USA) (Cat. No; K464-100)

### Enzyme-Linked Immunosorbent Assay Determination of the Renal Level of ERK1/2, HO-1, PI3K, IL-6, TNF-α, IκBα, NF-κB/p65, and Cleaved Caspase-3

The following parameters’ concentration was determined in renal tissue using demonstrated enzyme-linked immunosorbent assay (ELISA) kits following the manufacturers’ protocols. Phosphorylated ERK1/2 and Total ERK1/2 ELISA kits were obtained (Abcam, Cambridge, United Kingdom) (Cat. no; ab176660). Then, antibody mix was added to samples, and TMB substrate was added and catalyzed by horseradish peroxidase (HRP), generating blue coloration. This reaction was stopped by adding a stop solution, and the color intensity was measured at 450 nm. IκBα ELISA Kits were obtained from ELISA Genie (Dublin, Ireland) (Cat. No; RTFI00897); a biotin-detection antibody and TMB substrate were added after the stop solution. Optical density absorbance was read at 450 nm. Heme oxygenase-1 (HO-1) ELISA Kit was bought from BioVision incorporated (Milpitas, CA, USA) (Cat. no; E4525-100); biotin-detection antibodies were added to samples, and then 90-μl TMB was added and incubated (37 °C in the dark for 15–30 min). Shades of blue were seen and the stop solution was added; the result was read at 450 nm. Interleukin 6 ELISA Kit was acquired from Life Span BioSciences Inc (Seattle, WA, USA) (Cat. no; LS-F25921) for the assay based on double antibody sandwich method with chemiluminescence detection. The supplied plate has been pre-coated with a target-specific capture antibody. Samples and a biotin-conjugated detection antibody were added. An Avidin- HRP conjugate was then bound to the biotin, and unbound Avidin-HRP conjugate was washed away. A chemiluminescent substrate was then added, and the relative light units of each well were measured. PI3K ELISA Kits were obtained from MyBioSource (San Diego, California, United States) (Cat. no; MBS 702819); PI3K ELISA kit used double-sandwich ELISA technique. In which samples and biotin labeling antibody were added followed by Avidin-peroxidase conjugate addition. TMB substrate was used for color development, and the optical density was detected at 450 nm. TNF-α ELISA Kits were obtained from MyBioSource (San Diego, California, United States) (Cat. no; MBS355371); Biotin-antibody was added to samples, and HRP-avidin was added to each well, then TMB Substrate was added after washing, and finally 50 μl of stop solution was added. The optical density was determined at 450 nm. Cleaved caspase-3 ELISA Kits were obtained from MyBioSource (San Diego, California, United States) (Cat. no; MBS7244630) for the polyclonal anti-A-caspase-3 antibody and an A-caspase-3-HRP conjugate. The wells were then incubated with a HRP enzyme substrate to form a blue complex. The intensity of the color is measured at 450 nm.

NF-κB p65 ELISA Kit was obtained from Novus Biologicals, LLC (Littleton, Colorado, United States) (Cat. no; NBP2-29661); captured antibodies were added to each well and incubated overnight at 4 °C. Blocking buffer was added and incubated for 30 min. Positive and negative controls and samples were used, and the detecting antibody was added after washing. This is followed by diluted secondary antibody addition. After washing, pNPP substrate was added, and the color developed was detected at 405 nm.

### Histological Staining

Kidney tissue of different groups were fixed with 10% formalin saline at room temperature and embedded in paraffin. 4-μm thick sections of kidney tissue were cut and stained with hematoxylin and eosin (H & E) to determine morphological changes in the kidney. Additionally, kidney sections were subjected to PAS staining and scored under a microscope. Finally, renal tissue images were captured under a light microscope.

### Quantitative Real-time PCR for KIM-1, Nrf-2, and AKT Determination

Total RNA was isolated from the homogenized kidney tissue with a total RNA purification kit provided by Jena Bioscience (Munich, Germany) and stored at −80 °C. RNA was converted into complementary DNA by a cDNA archive kit (Applied Biosystems, Foster City, California, USA). qPCR was performed using GoTaq PCR master mix (Promega Co., Madison, USA). A protocol that included an initial denaturation step at 95 °C for 10 min, followed by 40 cycles of denaturing at 95 °C for 15 s, annealing and extension at 60 °C for 1 min then 60 °C for 30 s were performed on a StepOne Real-Time PCR System (Applied Biosystems, Foster City, California, USA). Descriptions of the sequences of oligonucleotide primers used is shown in Table 1

**Table 1:** Primer sequences used in this study were as follows

Markers (gene bank accession number)	Primer sequence (5′ to 3′)
KIM-1 (AF035963.1)	5′-TTTGGATCTGTACCCAGTGCTT-3′(sense),
	5′-CAAGGCCAGCCCTCTAATGG-3′ (antisense
Nrf-2 (NM_031789.2)	5′-TTGTAGATGACCATGAGTCGC-3′(sense),
	5′-TGTCCTGCTGTATGCTGCTT-3′ (antisense)
AKT (NM_033230.2)	5′-GCCCAACACCTTCATCATCC-3′(sense),
	5′-:GTCTCCTCCTCCTGCCGTTT-3′ (antisense)
β-actin (NM001106409.1) as an internal control	5′-GACGAGGCCCAGAGCAAGAGAGG-3′ (sense)
	5′-CTGCTTGCTGATCCACATCTGCT-3′ (antisense)

*KIM* kidney injury molecule -1, *Nrf2* nuclear factor erythroid 2-related factor 2, *AKT* protein kinase B

### Statistical Analysis

All data are presented as the mean ± standard deviation (SD). Between-group differences were detected via one-way analysis of variance. Finally, these data were analyzed using GraphPad Prism v.7.0 (GraphPad Software, Inc., San Diego, CA). *P* < 0.05 indicated statistical significance.

## Results

### Celastrol Enhanced Kidney Function and Renal Oxidative Stress

As shown in Table 2, IRI caused kidney function deterioration indicated by a significant increase in serum creatinine and BUN level compared with the sham group (*P* < 0.001). Also, renal IRI induced a significant increase in malondialdehyde (MDA) level and a remarkable decrease in reduced glutathione (GSH) level compared with sham-operated rats (*P* < 0.001). However, celastrol pretreatment significantly decreased serum creatinine and BUN levels, reduced the elevated MDA level, restored the depleted GSH level, and compared with the renal IRI group (*P* < 0.001).

**Table 2:** Effect of celastrol treatment (2 mg/kg; P.O) on serum creatinine, BUN, renal MDA and reduced glutathione levels in renal IRI rats

	Sham	Sham + cela	IRI	IRI + cela
Creatinine (mg/dl)	0.838 ± 0.19	0.88 ± 0.14	3.436 ± 0.066^+++^	1.584 ± 0.17^+++***^
BUN (mg/dl)	10.52 ± 3.11	17.22 ± 3.87	67.81 ± 12.16^+++^	34.98 ± 8.03^+++***^
MDA (µM/mg tissue protein)	0.565 ± 0.12	0.692 ± 0.09	3.245 ± 0.41^+++^	1.158 ± 0.19^++***^
Reduced GSH (µM/mg tissue protein)	404.2 ± 50.2	364.5 ± 57.6	150 ± 28.1^+++^	302.7 ± 50.2^++***^

All results were expressed as mean ± SD; *n* = 6 each

*MDA* malondialdehyde, *GSH* glutathione, *BUN* blood urea nitrogen, *IRI* ischemia reperfusion injury

^+++^*P* < 0.001, ^++^*P* < 0.01 compared with sham group and ^***^P < 0.001 compared with renal IRI group

### Celastrol Enhanced Nrf2 Gene Expression and HO-1 Level and Suppressed KIM-1, Gene Expression

**Fig. 2:**
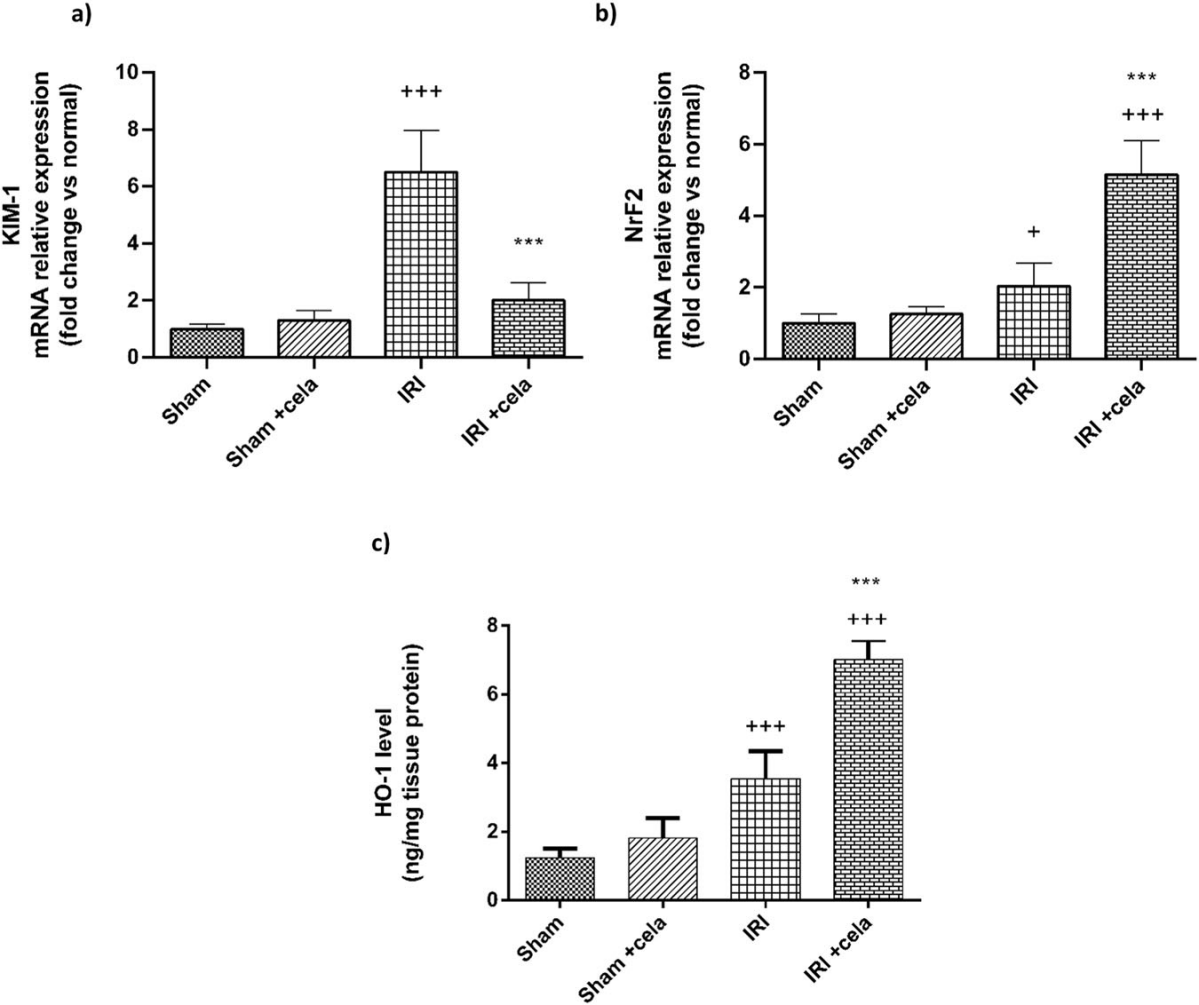
Effect of celastrol treatment (2 mg/kg; PO) on gene expression of **a** KIM-1, **b** Nrf2, and **c** HO-1 levels in renal IRI rats. All results were expressed as mean ± SD; *n* = 6 in each. KIM-1 kidney injury molecule-1, Nrf2 nuclear factor erythroid 2-related factor 2, HO-1 hemoxygenase-1, IRI ischemia-reperfusion injury. ^+++^*P* < 0.001, ^+^*P* < 0.05 compared with the sham group, ^***^P < 0.001 compared with the renal IRI group

Renal ischemia-reperfusion caused severe kidney damage confirmed by the significant upregulation of KIM-1 gene expression compared with sham-operated rats (*P* < 0.001). This is accompanied by enhancement of Nrf2 gene expression and rise in renal HO-1 level as a self-protective response to the injury. However, celastrol pretreatment displayed a notable renal protective and antioxidant effect by significant suppression of KIM-1 gene expression and remarkable upregulation of Nrf2 gene expression and subsequent elevation of HO-1 levels compared with the renal IRI group (*P* < 0.001) (Fig. 2a–c).

### Celastrol Activated PI3K/AKT Signaling

**Fig. 3:**
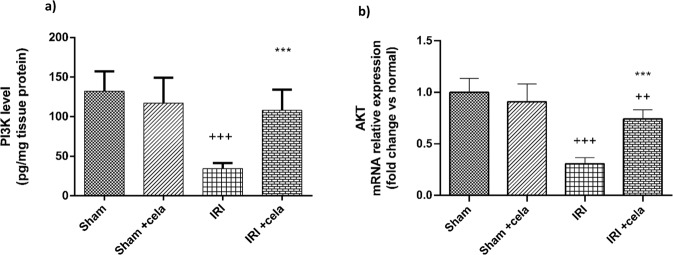
Effect of celastrol treatment (2 mg/kg; PO) on the level of **a** PI3K and gene expression of **b** AKT in renal IRI rats. All results were expressed as mean ± SD; *n* = 6 in each. PI3K phosphoinositide 3-kinase, AKT protein kinase B, IRI ischemia-reperfusion injury. ^+++^*P* < 0.001, ^++^*P* < 0.01 compared with the sham group, ^***^P < 0.001 compared with the renal IRI group

Ischemic injury triggered a significant suppression in cell survival mediating pathway; PI3K/AKT via a remarkable decrease in PI3K level and AKT gene expression (*P* < 0.001). Notably, celastrol treatment activates the PI3K/AKT signaling pathway through PI3K-level preservation and AKT gene expression enhancement compared with the IRI group (*P* < 0.001) (Fig. 3a, b).

### Celastrol Enhanced IκBα Level and Suppressed p-ERK1/2, t-ERK1/2, NFκBp65 Levels

**Fig. 4:**
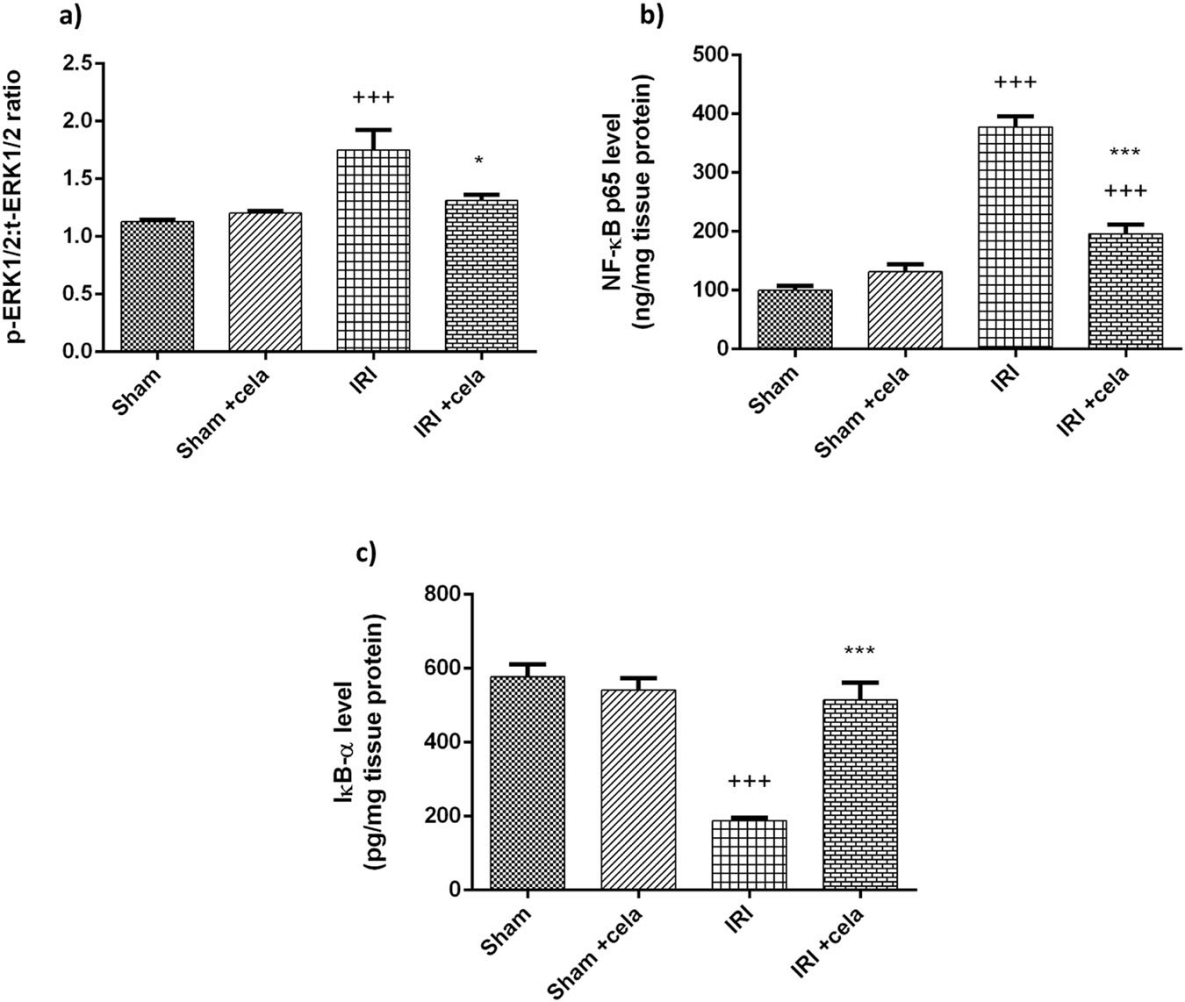
Effect of celastrol treatment (2 mg/kg; PO) on **a** P-ERK1/2:t-ERK1/2 ratio, levels of **b** NF-kBP65 and **c** IκB-α in renal IRI rats. All results were expressed as mean ± SD; *n* = 6 each. ERK extracellular signal-regulated kinase, NF-kBP65 nuclear transcription factor-kappa B P65, IκB-α inhibitor of kappa B alpha, IRI ischemia-reperfusion injury. ^+++^*P* < 0.001 and ^++^*P* < 0.01 compared with the sham group, ^***^P < 0.001 and *P < 0.05 compared with the renal IRI group

Kidney injury induced by ischemia-reperfusion significantly enhanced ERK 1/2 phosphorylation and increased p-ERK: t- ERK ratio compared with the sham group (*P* < 0.001). Moreover, renal IRI rats exhibited a significant increase in NFκBp65 with significant suppression of NfκB inhibitor protein; IκBα levels (*P* < 0.001). These findings were reversed upon celastrol treatment; p-ERK: t- ERK ratio and NFκBp65 level were significantly decreased (*P* < 0.05 and *P* < 0.001, respectively) with a remarkable increase in IκBα level compared with the renal IRI group (*P* < 0.001) (Fig. 4a–c).

### Celastrol Suppressed IL-6, TNFα, and Cleaved Caspase-3 Levels

**Fig. 5:**
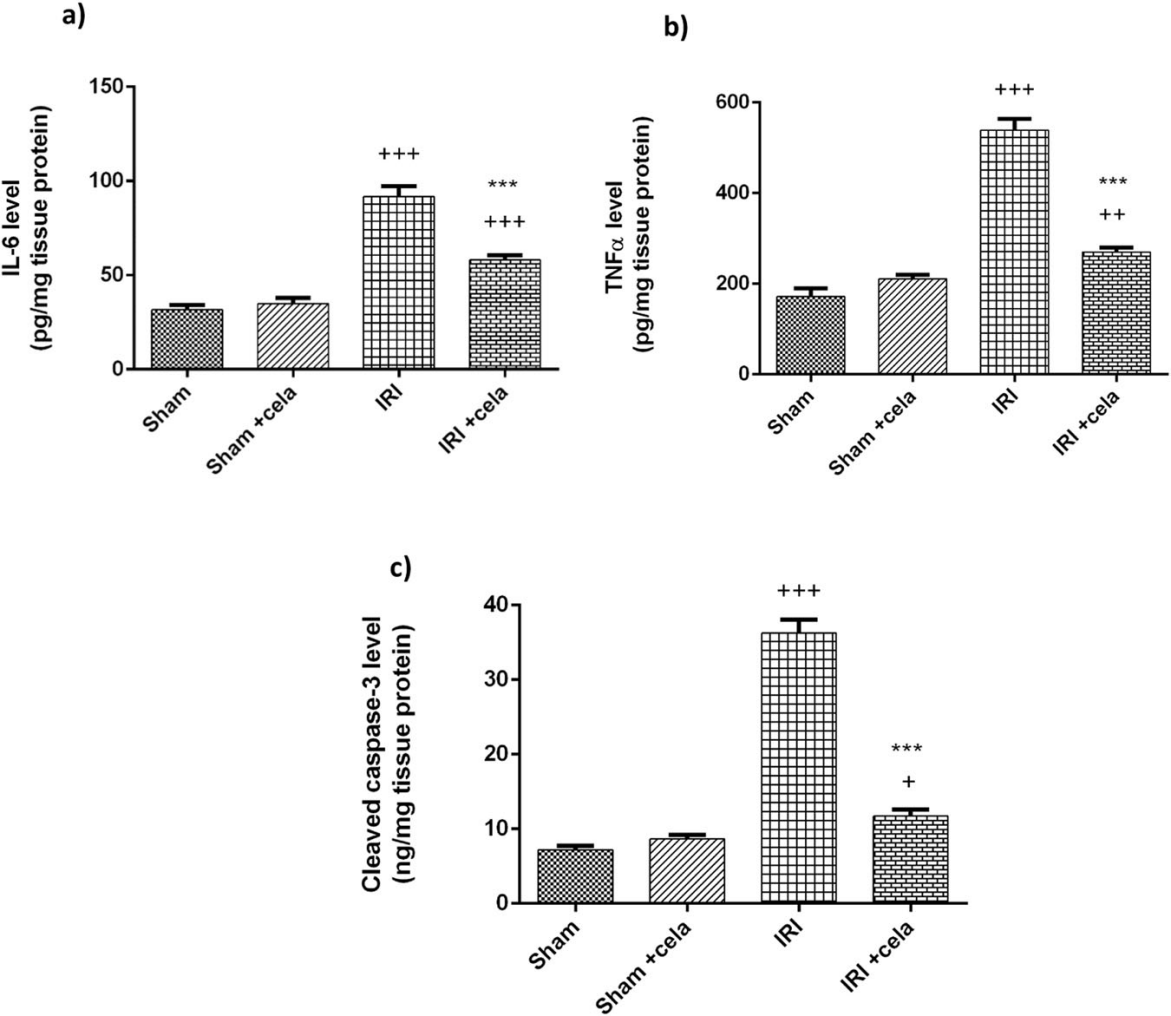
Effect of celastrol treatment (2 mg/kg; PO) on levels of **a** IL-6, **b** TNFα, and **c** cleaved caspase-3 in renal IRI rats. All results were expressed as mean ± SD; *n* = 6 each. IL-6 interleukin-6, TNF α tumor necrosis factor-alpha, IRI ischemia-reperfusion injury. ^+++^*P* < 0.001, ^+^*P* < 0.05 compared with the sham group, ***P < 0.001 compared with the renal IRI group

As presented in (Fig. 5a–c), kidney injury was accompanied by a significant increase in inflammatory cytokines, IL-6 and TNFα levels (*P* < 0.001). Also, ischemia-reperfusion significantly increases the apoptotic marker cleaved caspase-3 level compared with sham-operated rats (*P* < 0.001). Celastrol treatment exhibited a pronounced anti-inflammatory and antiapoptotic effect shown by the remarkable decrease in IL-6, TNFα, and cleaved caspase-3 levels compared with the renal IRI group (*P* < 0.001).

### Celastrol Improved Histological Alterations Induced via Renal Ischemia-reperfusion

**Fig. 6:**
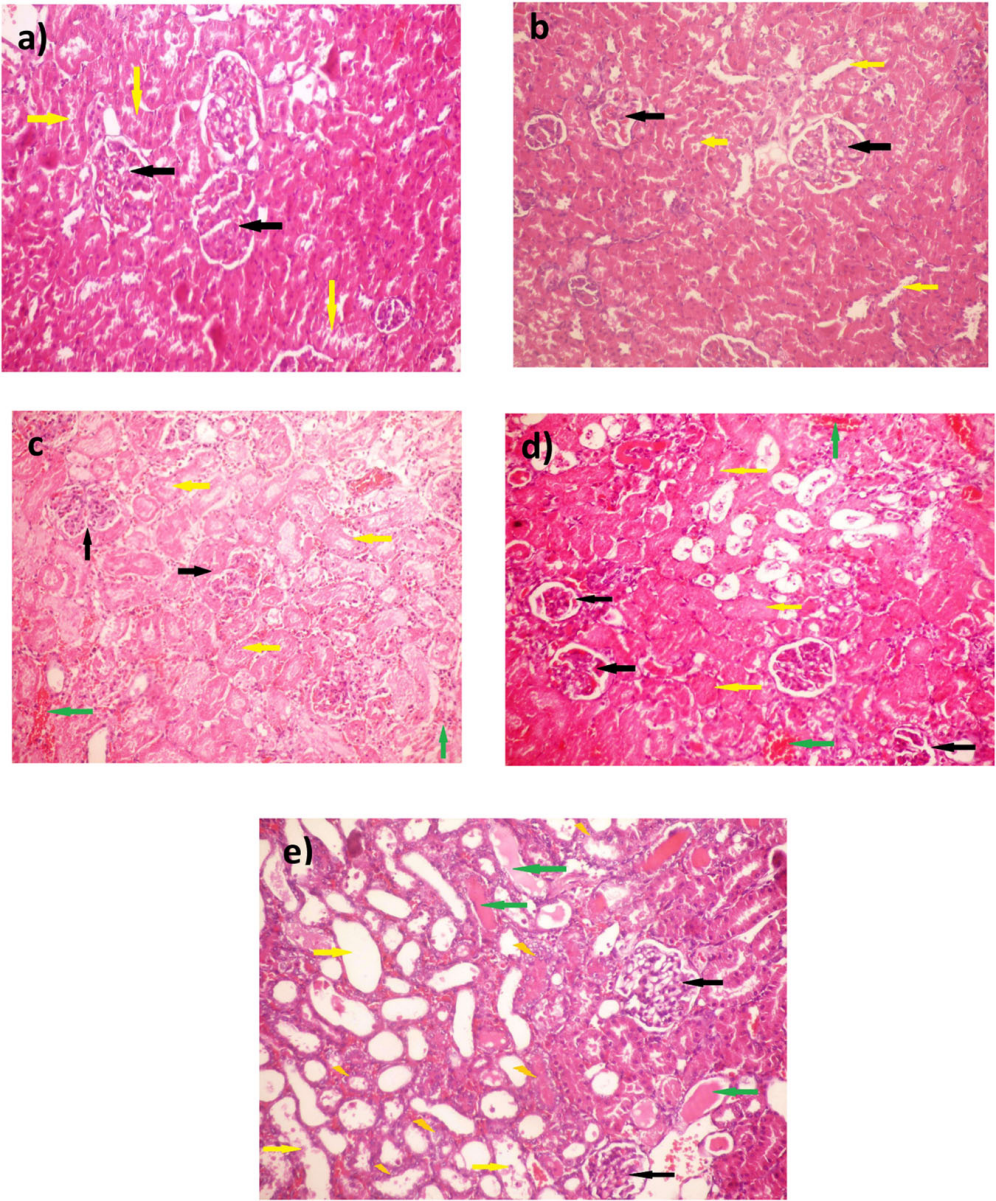
Effect of celastrol treatment (2 mg/kg; PO) on H&E-stained kidney sections of renal IRI rats. Representative microscopic pictures of H&E-stained kidney sections showed well-organized glomeruli (black arrows) and normal tubules (yellow arrow) in sham-operated rats (**a**). Kidney sections from sham-operated rats treated with cela showed mild disorganization of the glomeruli with moderate congestion of its capillaries (black arrows), and the tubules are within normal (yellow arrows) (**b**). In kidney sections of renal IRI rats, most glomeruli were collapsed with focal necrosis (black arrows), the tubules showed marked necrosis with loss of the histologic details (yellow arrows), and the stroma showed many foci of interstitial hemorrhage (green arrows) (**c**, **d**). Kidney sections from renal IRI + cela treated rats showed Hyperplastic glomeruli with mild congestion (black arrows), some tubules showed sloughed epithelial cells (yellow arrows), others showed hyaline casts (green arrows), regenerated tubules with active vesicular nuclei are detected between damaged tubules (crooked arrows) (**e**) (H&E ×200). H&E hematoxylin and eosin, cela celastrol, IRI ischemia-reperfusion injury

Kidney sections from sham-operated rats stained with H and E stain showed well-organized glomeruli (black arrows) and normal tubules (yellow arrow) (Fig. 6a). Additionally, sham-operated rats treated with cela showed mild disorganization of glomeruli and moderate congestion of its capillaries (black arrows), and the tubules are within normal (yellow arrows) (Fig. 6b). However, kidney sections from renal IRI rats displayed collapsed glomeruli with focal necrosis (black arrows), and the tubules showed marked necrosis with loss of the histologic details (yellow arrows). In addition, the stroma showed many foci of interstitial hemorrhage (green arrows) (Fig. 6c, d). Celastrol-treated rats’ kidneys showed hyperplastic glomeruli with mild congestion (black arrows), some tubules showed sloughed epithelial cells (yellow arrows), and others showed hyaline casts (green arrows). Besides, regenerated tubules with active vesicular nuclei are detected between damaged tubules (crooked arrows) (Fig. 6e).

**Fig. 7:**
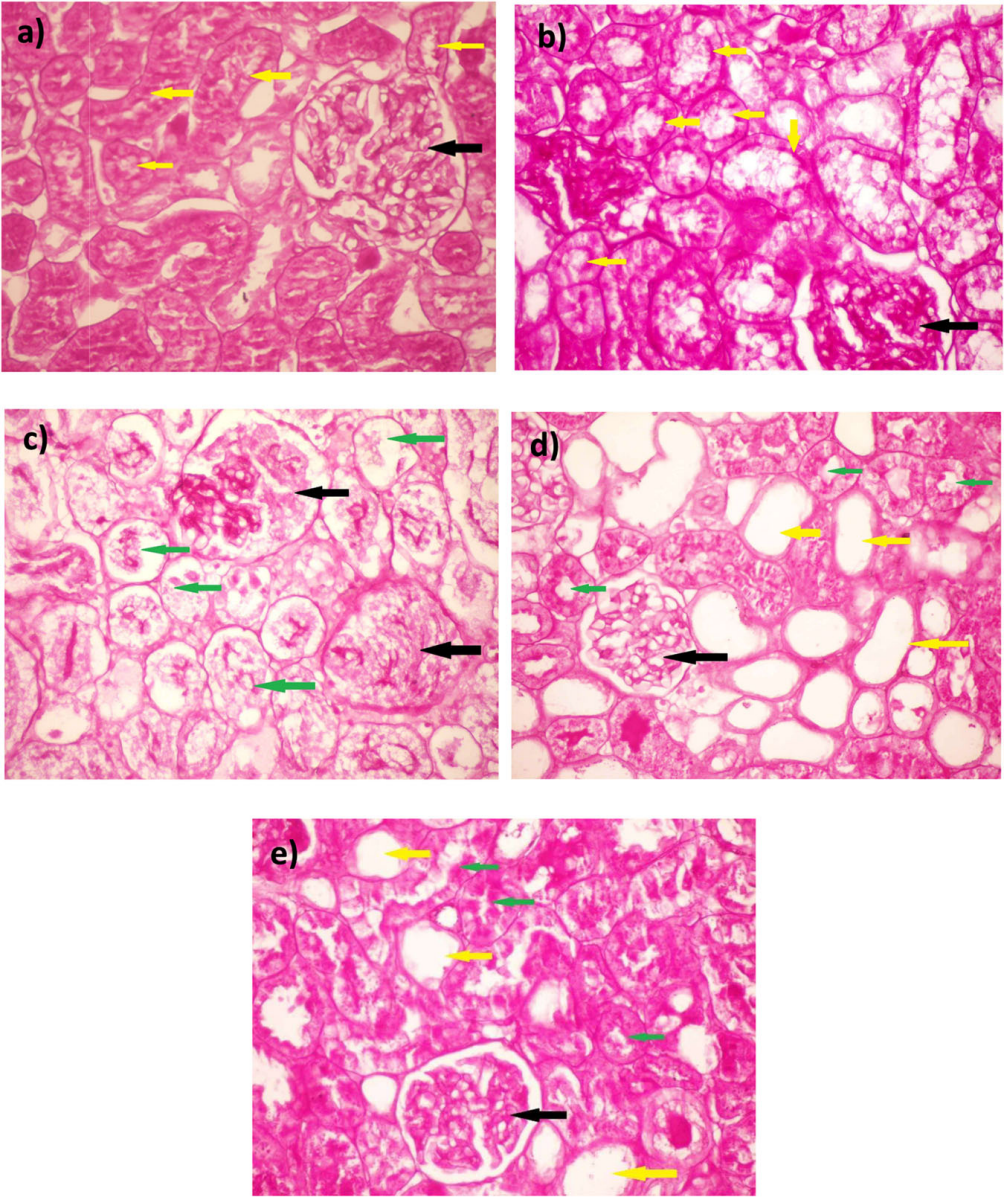
Effect of celastrol treatment (2 mg/kg; PO) on PAS-stained kidney sections of renal IRI rats. Representative microscopic pictures of PAS-stained kidney sections showed well-organized glomeruli with good staining of the capillary’s basement membrane with PAS stain (black arrow). In addition, the tubules showed well-demarcated outer basement membrane and inner brush border (yellow arrows) in sham-operated rats (**a**). Kidney sections from sham-operated rats treated with cela showed PAS staining of the glomerular capillaries’ basement membrane (black arrows) with prominent PAS-positive brush border (yellow arrows) (**b**). kidney sections of renal IRI rats showed wrinkling and thickening of capillary basement membranes and collapse of the capillary lumen (black arrows) with significant tubular necrosis and cast formation (green arrows) (**c**). Kidney sections from renal IRI + cela treated rats showed hyperplastic glomeruli with the moderate demarcation of its capillaries’ basement membrane (black arrow). Damaged tubules lost their basement membrane and inner brush border (yellow arrows). Regenerated tubules with the restoration of PAS staining of the basement membrane and brush border (green arrows) (**d**, **e**) (PAS stain ×400). PAS Periodic acid–Schiff, cela celastrol, IRI ischemia-reperfusion injury

Kidney sections from sham-operated rats stained with PAS stain showed well-organized glomeruli with good staining of the capillary’s basement membrane with PAS stain (black arrow), and the tubules showed well-demarcated outer basement membrane and inner brush border (yellow arrows) (Fig. 7a). Also, kidney sections from sham-operated rats treated with cela showed PAS staining of the glomerular capillary basement membrane (black arrows) with a prominent PAS-positive brush border (yellow arrows) (Fig. 7b). Renal IRI rat’s kidney showed wrinkling and thickening of capillary basement membranes and collapse of the capillary lumen (black arrows) with significant tubular necrosis and cast formation (green arrows) (Fig. 7c). Kidney sections from IRI + cela treated rats showed hyperplastic glomeruli with the moderate demarcation of its capillaries’ basement membrane (black arrow). Additionally, there were damaged tubules with loss of basement membrane and inner brush border (yellow arrows) and regenerated tubules with restoration of PAS staining of the basement membrane and brush border (green arrows) (Fig. 7d, e).

## Discussion

Celastrol has been studied for its protective effect against renal IRI in rats by inhibiting NF-kB activation [25]. However, in this study, we investigated the antioxidant potential of celastrol through its effect on Nrf2/HO-1 pathway. Furthermore, we explored celastrol’s effect on PI3K/AKT cell survival pathway, ERK phosphorylation, NF-kB activation, and subsequent inflammatory and apoptotic responses.

Our study revealed a significant decrease in MDA level and a significant increase in reduced GSH content in celastrol-treated rats compared with renal IRI rats. Renal IR is characterized by a massive ROS production that derives kidney cell damage and apoptosis [26]. Celastrol could preserve kidney cells from damage and correct the deteriorated kidney functions; serum creatinine and BUN in renal IR rats result from oxidative stress inhibition. The celastrol protective effect against renal injury was also proved by the significant downregulation of kidney injury molecule (KIM-1) gene expression compared with the IRI group. Histopathological observations confirmed these biochemical results. These results follow previous reports that proved celastrol’s antioxidant activity in other kidney injury rat models [27, 28]. We tried underlining the possible mechanisms by which celastrol may exert its antioxidant potential by exploring the celastrol effect on Nrf2/HO-1 pathway.

Nrf2 is a transcription factor that plays a protective role in experimental acute kidney injury through regulation of endogenous antioxidant enzyme gene expression and subsequent alleviation of oxidative stress [29]. Nrf2^−/−^ transgenic mice showed provoked kidney injury after IRI [30]. As well, Nrf2 is considered a key regulator of HO-1 gene expression [31]. HO-1 represents a protective system by targeting the pro-oxidant substrate; heme in a reaction generates the antioxidants biliverdin and bilirubin and the vasodilator molecule; carbon monoxide with antiapoptotic properties [32]. Our results showed an enhancement of Nrf2 gene expression and elevation in HO-1 levels in IRI rats as a defensive response to IR injury, and these results follow a previous study [33]. Meanwhile, celastrol-treated rats exhibited a significant upregulation of Nrf2 gene expression along with a significant increase in HO-1 renal level compared with renal IRI rats, indicating that celastrol exerts its protective role against renal IRI by activating Nrf2 with a subsequent increase in HO-1 renal level.

Our results suggested the protective effect of celastrol in renal IRI model via activation of the cell survival mediating pathway; PI3K/AKT indicated by a significant increase in PI3K renal level and remarkable upregulation of AKT gene expression in the ecelastrol-pretreated group compared with the IRI group. PI3K/AKT signaling pathway plays a significant role in protecting kidney cells against IRI [34, 35]. This protective effect has been attributed to its inhibitory effect on many inflammatory cytokines and pro-apoptotic mediators that evolved in response to kidney injury [36, 37]. Interestingly, it was reported that the PI3K/AKT pathway mediates the activation of Nrf2 nuclear translocation and subsequent HO-1 induction [38, 39]. Our results may provide further evidence that celastrol exerts its reno-protective effect by activating the Nrf2/HO-1 pathway through the PI3K/AKT-dependent mechanism.

An ERK, one of the MAPK family members and NF-κB transcription factors, is involved in renal IRI. Their activation is considered a key for inflammatory response and cell apoptosis regulation during the IR process [40, 41]. Furthermore, it has been reported that NF-κΒ p65 subunit phosphorylation and acetylation are central in NF-κΒ activation [42]. Therefore, we evaluated the phosphorylated, total ERK1/2, NF-kBP65, and the NF-kB inhibitor protein (IκB-α) levels to estimate the role of celastrol in the modulation of MAPK and NF-kB signaling pathways and subsequent inflammatory cytokine (IL-6 and TNFα) regulation. In addition, the key regulatory factor in apoptosis; cleaved caspase-3 level, was also determined. Remarkably, celastrol pretreatment displayed a notable inhibition of ERK 1/2 phosphorylation accompanied by a significant decrease in NF-kBP65 and an increase in IκB-α levels with a subsequent decrease in IL-6, TNFα, and cleaved caspase-3 levels compared with the renal IRI group.

Stimulation of MAPK signaling pathway comprising ERK1/2 phosphorylation activation is initiated by diverse inflammatory and stressful stimuli and is reported to be associated with inflammation and cell death during renal injury [43]. MAPK activation may also be closely related to NF-κB translocation to the nucleus and subsequent activation of various inflammatory and apoptotic cascades [44–46]. The master kinase in the toll-like receptor pathway and activated transforming growth factor beta-activated kinase 1 (TAK1) mediates IKK activation, which is essential in NF-κB activation. Likewise, TAK1 mediates the activation of MAPKs [47]. This suggests interesting crosstalk between MAPK signaling and the NF-κB pathway. Along this line, studies have emphasized celastrol’s inhibitory effect on MAPKs and NF-κB pathway, and thus suppression of inflammatory cytokines and pro-apoptotic mediators release in different rat models [48–51]. Consistently, our results showed that celastrol pretreatment possibly exerts its protective effect against IRI through inhibition of ERK phosphorylation and NF-κB mediated inflammation and apoptosis.

## Conclusion

Celastrol pretreatment showed a reno-protective potential against renal IRI by suppressing oxidative stress by enhancing the antioxidant defense system; Nrf2/HO-1. Also, celastrol showed activation of the survival signaling pathway; PI3K/AKT that protects renal tissue from IRI. In addition, Celastrol also displayed a significant anti-inflammatory and antiapoptotic effect through the inhibition of NF-κB activation and ERK phosphorylation, which suppressed their downstream effectors, inflammatory cytokines (IL-6 and TNFα) and pro-apoptotic mediator (caspase-3). These findings prove celastrol is a promising candidate for protecting the kidney against IR injury with potential clinical application.

## Data Availability

The authors confirm that the data supporting the findings of this study are available upon request.

## Abbreviation

AKT: Protein kinase B
ERK: Extracellular signal-regulated kinase
HO-1: Heme oxygenase 1
IL-6: Interleukin-6
IRI: Ischemia-reperfusion injury
KIM-1: Kidney injury molecule
NF-κB: Nuclear factor-kappa B
Nrf2: Nuclear factor erythroid 2-related factor 2
PI3K: Phosphoinositide 3-kinase
TNF-α: Tumor necrosis alpha
